# Biochemical Pregnancy During Assisted Conception: A Little Bit Pregnant

**DOI:** 10.4021/jocmr1008w

**Published:** 2013-06-21

**Authors:** John Jude Kweku Annan, Anil Gudi, Priya Bhide, Amit Shah, Roy Homburg

**Affiliations:** a Homerton Fertility Centre, Homerton University Hospital NHS Foundation Trust, Homerton Row, E9 6SR, London, UK

**Keywords:** Biochemical pregnancy, In vitro fertilisation, Assisted reproductive technology, Beta human chorionic gonadotropin, Embryo transfer, Intracytoplasmic sperm injection

## Abstract

Assisted reproductive technology (ART) has revolutionized the management of subfertility as many couples who previously had no hope of achieving a pregnancy are able to do so. Several factors contribute to the successful outcome of assisted conception. The period of waiting for the pregnancy test after assisted conception could be very crucial to the patient. One outcome of assisted conception could be a positive pregnancy test which could lead to a clinical pregnancy resulting in a live birth, clinical pregnancy resulting in a miscarriage or a biochemical pregnancy. A negative pregnancy test, failure to fertilise and failure to respond to stimulation usually lead to a big blow to the couple. As far as biochemical pregnancy is concerned, its exact aetiology remains unknown. There are no definite predictive factors for its occurrence that can be remedied in subsequent cycles. Several associated aetiologies have been suggested in the literature. This review aims at addressing the issue of biochemical pregnancy after assisted conception as a prelude to conducting further studies to assess if there are any predictive factors for its occurrence.

## Introduction

With the advent of assisted reproductive technology (ART), many couples who previously had no hope of achieving a pregnancy are able to do so. The various stages involved in ART potentially exert a significant psychological, emotional, physical and (in most cases) financial impact on the couple.

After embryo transfer, the couple have to wait for the result of the serum beta human chorionic gonadotropin (β-hCG) pregnancy test. This could be associated with significant psychological morbidity as it represents the first decisive hurdle that must be confronted.

One of the possible outcomes of the pregnancy test is a biochemical pregnancy; where the initial pregnancy test is positive but does not progress into a clinical pregnancy.

A biochemical pregnancy sounds like a ‘false positive pregnancy test’; as if the patient was not really pregnant at all. To the patient, this becomes a conundrum! The question that would usually arise from the patient is ‘How can I be a “little bit” pregnant?’ The truth is that a biochemical pregnancy was indeed a conception and is actually a very early miscarriage.

The advent of high-sensitivity pregnancy tests has now made early diagnosis of pregnancy widely possible. A pregnancy test can be positive as early as the first days of the approximate time of implantation or when traces of β-hCG are detectable in the maternal serum. It has been established that as many as 25% of pregnancies fail even before the woman has any subjective indication that she is pregnant, that is, before she misses her menstrual period or has symptoms of pregnancy. In the general population, most biochemical pregnancies go unrecognized. The recognizable ones are just a tip of the iceberg. Biochemical pregnancies are diagnosed under active monitoring for pregnancy when β-hCG levels are tested prior to a missed menstrual period but may occur spontaneously following a missed period.

The American Society of Reproductive Medicine and the Society for Assisted Reproductive Technology distinguish biochemical pregnancies from clinical pregnancies, which include spontaneous miscarriages. The transient rise in β-hCG that characterizes a biochemical pregnancy is distinct from the widely recognized outcomes of a clinical pregnancy, which include spontaneous and induced miscarriages, ectopic pregnancy, and delivery. In the absence of routine use of ultrasound, a biochemical pregnancy could be defined by the combination of a low peak in β-hCG (< 100 mIU/mL), rapid fall in urinary or serum β-hCG concentration, and lack of substantial delay in onset of the next menstrual period to help differentiate this entity from a clinical pregnancy [1].

The objectives of this review are to address biochemical pregnancy, discuss some of the aetiological associations and discuss its significance as far as subsequent assisted conception cycles are concerned. This is aimed to be a prelude to looking in-depth into investigating for any potential predictive factors that could assist practitioners to predict those patients likely to have a biochemical pregnancy.

## Synonyms

Various terminologies have been used by different authors to describe a biochemical pregnancy. Biochemical pregnancy has been described using various terminologies [2]. 1). ‘trophoblast in regression’; 2). ‘pre-clinical embryo loss’; 3). ‘chemical pregnancy’.

## Definitions

Biochemical pregnancy has been variously defined by different authors. All these definitions express the same idea. Sher defined a “chemical pregnancy” as one where in spite of the beta hCG test being “positive”, the pregnancy fails to progress to the point of ultrasound confirmation [3]. This implies that a chemical pregnancy is a very early pregnancy loss, characterized by a positive pregnancy test (β-hCG level) which, however, is not maintained. Moreover such a pregnancy never reaches the stage where a gestational sac is seen on ultrasound examination. Therefore, the name “chemical” pregnancy, since the gestation is diagnosed only by chemical means. In contrast, a so-called clinical pregnancy is characterized by the fact that it has reached a stage where the gestation can be seen on ultrasound examination.

According to Winter et al, in assisted reproductive technology (ART), pregnancy loss before clinical detection by ultrasound scan is commonly referred to as biochemical pregnancy [4]. Additionally, the term chemical pregnancy has been used to describe a transiently positive β-hCG level not associated with the development of an embryo or even a gestational sac [1].

A similar idea was expressed in the paper on ‘Normal and abnormal implantation in spontaneous in-vivo and in-vitro human pregnancies’. The authors defined biochemical pregnancy as pregnancy loss too early for any corroborative clinical sign, so the only evidence of implantation is a higher than normal β-hCG concentration [5].

In this vein, De Neubourg et al, in their study of single top quality embryo transfer, defined biochemical pregnancy using β-hCG level as two increasing values of β-hCG > 5 IU/L. They included biochemical pregnancy with first trimester pregnancy loss (FTPL) [6]. FTPL is a pregnancy leading to pregnancy loss prior to 13 weeks of gestation (namely biochemical pregnancy, clinical miscarriage and ectopic pregnancy).

## Mechanism of Biochemical Pregnancy

Women treated by ART are routinely monitored for early detection of pregnancy by measuring serum β-hCG concentration on a specific day, usually 14 - 17 days following oocyte retrieval, equivalent to ovulation in the general population, and again by ultrasound scan at about 6 - 7 weeks gestation [4].

After embryo transfer, the developing embryo begins to secrete β-hCG. Once the embryo begins to implant and there is trophoblastic invasion into the decidua, more human chorionic gonadotropin (β-hCG) gets released into the recipient’s blood stream. If enough β-hCG is produced by the embryo, this can be detected on the β-hCG blood test.

About 12 days after oocyte retrieval, 9 days after a day-3 embryo transfer and 7 days after a blastocyst transfer, the woman should have a quantitative β-hCG blood pregnancy test performed. By that time almost all β-hCG trigger injected to prepare the developing oocytes for retrieval should have disappeared from the woman’s bloodstream. Thus the detection of > 5 IU of β-hCG per ml of blood tested is an indication that the embryo has attempted/begun to implant. However, since with third-party IVF (namely ovum donation, embryo adoption) or frozen embryo transfers, no β-hCG “trigger shot” is administered, the detection of any amount of β-hCG in the blood is regarded as significant [3].

However, because the pregnancy does not develop normally, the β-hCG levels decline and no pregnancy sac can be seen on ultrasound scanning. As such this pregnancy is only diagnosed biochemically.

## β-hCG Cut-Offs for Diagnosing Biochemical Pregnancy

Different β-hCG cut-offs have been suggested by various authors for biochemical pregnancy.

Schreiber et al used the combination of a low peak in β-hCG (< 100 mIU/mL), rapid fall in urinary or serum β-hCG concentration, and lack of substantial delay in onset of the next menstrual period to help differentiate biochemical pregnancy from a clinical pregnancy [1].

De Neubourg et al, in their study of single top quality embryo transfer, defined biochemical pregnancy with a cut-off as two increasing values of β-hCG > 5 IU/L [6].

Biochemical pregnancy was subdivided by other authors according to the maximum β-hCG concentrations attained, for example, β-hCG maximum between 40 and 150 IU/L, 10 and 39 IU/L or 5 and 9 IU/L. This implies their minimum cut-off was also 5 IU/L [5].

## Incidence

In spontaneous pregnancies, biochemical pregnancies are thought to be fairly common, involving as many as half of all pregnancies, but an accurate number is hard to determine because most women who experience a biochemical pregnancy never even realize they are pregnant unless they are trying to conceive and testing regularly and early. Many biochemical pregnancies are discovered today that would otherwise have gone undetected due to the ultra sensitive pregnancy tests on the market, which make it easier to get a positive result 3 or 4 days before a woman’s period is due.

Biochemical pregnancies are much more common than thought. In fact, between 50% and 60% of all first-time pregnancies are thought to end in miscarriage - a large majority of which can be attributed to biochemical pregnancies [3].

Additionally, it has been established that as many as 25% of pregnancies fail even before the woman has any subjective indication that she is pregnant, that is, before she misses her menstrual period or has symptoms of pregnancy [1].

De Neubourg et al conducted a study of a total of 370 single top quality embryo transfers in patients younger than 38 years of age. This resulted in 192 pregnancies (51.9%). Thirty cycles (8.1%) ended in a biochemical pregnancy, four (1.1%) cycles ended in an ectopic pregnancy, 23 (6.2%) cycles ended in a clinical miscarriage and 135 (36.5%) cycles resulted in ongoing pregnancies. A total of 57 (29.7%) pregnancies were lost [6].

In frozen embryo replacement cycles, Salumets et al reviewed the outcome of 1,242 frozen embryo transfers with respect to the age of the woman, the method of fertilization, embryo quality before and after freezing and the number of embryos transferred. They found that the pregnancy (positive hCG) and clinical pregnancy rates were 25.8 and 21.1%, respectively. A total of 107 (33.3%) of the 321 pregnancies identified by a positive hCG test miscarried either before (18.4%) or after (15%) the clinical recognition of gestational sac(s). They also found that increased woman’s age at IVF/ICSI treatment was the only parameter elevating the biochemical pregnancy rate [7].

Biochemical pregnancy and clinical abortion rates of 15-20% and 20-25%, respectively, have been reported after the transfers of cryopreserved embryos by various authors [8-11].

## Aetiological Associations

The exact aetiology of biochemical pregnancy after ART is unknown. However several associated factors have been reported in literature.

According to Sher, chemical pregnancies occur quite frequently following IVF. While they usually result from a chromosomally abnormal (aneuploid) embryo trying to implant, they can also be due to the uterine lining (for anatomical, immunologic or other reasons) being insufficiently receptive to allow healthy embryo implantation [3]. In order to assess the endometrium as an aetiological cause of biochemical pregnancy, Dickey et al assessed the degree of pre-ovulatory endometrial thickness. In order to assess the relationship between pre-ovulatory endometrial thickness and pattern and biochemical pregnancy, Dickey et al retrospectively analysed the pregnancy outcome in 81 patients undergoing ovulation induction evaluated by vaginal ultrasound on the day of human chorionic gonadotrophin (hCG) administration or luteinizing hormone (LH) surge. Biochemical pregnancies occurred in 7/32 (21.9%) pregnancies when endometrial thickness was < 9 mm, compared to 0/49 when endometrial thickness was ≥ 9 mm on the day of hCG administration or LH surge (P < 0.0025). Endometrial thickness was related to the cycle day of hCG or LH surge (r = 0.37, P < 0.001) but was unrelated to oestradiol level on the day of hCG administration or LH surge (r = 0.12). Biochemical pregnancies were related to endometrial pattern (r = -0.22, P = 0.02) but were unrelated to maternal age or previous abortions. Clinical abortions were related to age (r = 0.26, P = 0.01) and to previous abortion (r = 0.25, P = 0.013) but were unrelated to endometrial pattern. Neither biochemical pregnancy nor clinical abortion was related to oestradiol or LH levels on the day of hCG administration or LH surge. These findings suggest that the majority of biochemical pregnancies do not result from karyotypically abnormal embryos, as do clinical abortions [12].

However, an Italian study by Fachchinetti et al evaluated stress as an aetiological association of biochemical pregnancy. They conducted a controlled, prospective clinical study to evaluate the association between the vulnerability to stress and the treatment outcome of couples undergoing IVF-ET. Forty-nine infertile women were consecutively admitted to standard superovulation treatment. The mean age was 33.9 years and the mean duration of infertility was 6.3 years. Reasons for assisted reproduction were mechanical factor in 22 cases, sperm problem in 9 cases, and endocrine disorder in 6 cases. In 12 cases, infertility was unexplained. More than 55% already had an IVF-ET attempt. On the day of oocyte pick-up, subjects were submitted to Stroop Color and Word test, a task measuring the ability to cope with a cognitive stressor, involving attentional and sympathoadrenal systems. Systolic blood pressure (SBP) and diastolic blood pressure, as well as heart rate (HR) were measured at baseline, during the test, and 10 minutes after the end of testing. The main outcome measure(s) were the evidence of a biochemical pregnancy (β-hCG value 12 days after ET) define the success and failure groups. The results showed that sixteen women (33%) had a biochemical pregnancy, twelve (24%) also had ultrasound evidence of intra-uterine pregnancy and eight (16%) gave birth to healthy infants. Age, education, causes, and duration of infertility were similar in the success and failure groups. The latter were more involved in a job outside home than the former. Moreover, they had a lower number of both fertilized oocytes and transferred embryos. In response to the Stroop test, every subject reported an increase of cardiovascular parameters. However, women becoming pregnant showed a lower response of both systolic blood pressure and heart rate than women who failed. They concluded that both a major cardiovascular vulnerability to stress and working outside home are associated to a poor outcome of IVF-ET treatment [13].

The sperm has also been implicated as a cause of early pregnancy loss. Zini et al conducted a systematic review and meta-analysis of studies on sperm DNA damage and pregnancy loss after an IVF and/or ICSI pregnancy. Two by two tables were constructed and odds ratios (ORs) were derived from 11 estimates of pregnancy loss (five IVF and six ICSI studies from seven reports). These 11 studies involved 1549 cycles of treatment (808 IVF and 741 ICSI cycles) with 640 pregnancies (345 IVF and 295 ICSI) and 122 pregnancy losses. The combined OR of 2.48 (95% CI 1.52, 4.04, P < 0.0001) indicates that sperm DNA damage is predictive of pregnancy loss after IVF and ICSI. The conclusion of the authors was that sperm DNA damage is associated with a significantly increased risk of pregnancy loss after IVF and ICSI. These data provide a clinical indication for the evaluation of sperm DNA damage prior to IVF or ICSI and a rationale for further investigating the association between sperm DNA damage and pregnancy loss [14].

The finding of an association between sperm DNA damage and pregnancy loss is consistent with the results reported in another otherwise eligible study. Indeed, Virro et al also observed an increased pregnancy loss in IVF and IVF/ICSI pregnancies achieved using samples with DNA damage [15].

Although the possible mechanism(s) that underlie the association between sperm DNA damage and pregnancy loss are not known, animal studies indicate that sperm DNA damage can lead to abnormal embryo development and impaired embryo implantation [16-18].

It is becoming more and more clear that not only the genetic make-up of the oocyte but also the integrity of the meiotic spindle is pivotal to early embryogenesis. Recent hypotheses point to the presence of two distinct mechanisms of embryo wastage in early embryo development. The first is chaotic mosaicism which can be considered a non-nuclear (mitochondrial) mechanism of early embryo wastage. The second is nondisjunction which affects early embryo development through a nuclear (chromosomal) mechanism. Therefore, improvement of the success rate in IVF/ICSI should be directed to making the best possible embryo selection in order to improve the pregnancy rate and to decrease fetal wastage. It has been shown that aneuploidy screening in preimplantation embryos does not improve the implantation rate but reduces the embryo loss after implantation. The question of whether subfertile couples are more prone to pregnancy loss remains unsolved. No such association has been reported despite the chance of over-representation in this group.

In frozen embryo replacement cycles, Edgar et al proposed that although the reasons for impaired pregnancy and elevated spontaneous abortion rates following frozen embryo transfer are not completely understood, they are most likely caused by the damage to embryos occurring during the freezing and thawing procedures [19].

Additionally, in frozen embryo replacement cycles, the detailed analysis of different clinical and embryological factors that could possibly influence the probability for pregnancy loss before the clinical recognition of gestational sac(s) displayed that the biochemical pregnancy rate was solely determined by the woman’s age at embryo freezing. The calculated ORs for the biochemical pregnancy rate for women 5 years younger and 5 years older than women of the mean age were 0.79 (95% CI 0.67 - 0.98) and 2.08 (95% CI 1.21 - 3.56), respectively [7].

## Significance of Biochemical Pregnancy

Clearly, to the IVF patient, the diagnosis of a biochemical pregnancy represents a severe disappointment. However its occurrence provides clear evidence that at least one embryo reached the advanced preimplantation phase of development (the blastocyst stage), went on to “hatch” and attempted to implant. As such a biochemical pregnancy can often be regarded as being a “dark cloud that has a silver lining” because it offers the hope of a successful clinical pregnancy in the future [3].

The most difficult aspect of a biochemical pregnancy is the initial false hope that it brings - the excitement of finally becoming pregnant after a stressful treatment cycle - and then to deal with the devastating disappointment of not being pregnant. For many couples, this can be the last straw which breaks the camel’s back. They often find it easier to deal with a negative hCG result; rather than an initial hopeful positive result which then declines.

The commonest question patients ask after a biochemical pregnancy is - Why did this happen? Did I do something to harm the embryo? Does this mean I am never destined to have a baby? Does this mean my uterus is defective and is rejecting the baby? None of this is true! The fact that the hCG was positive means that the embryo implantation process did start, and this means that the prognosis for a healthy pregnancy in the future is actually better than for someone with a negative hCG. This is supported adequately in the literature.

De Neubourg et al concluded from their study of single top quality embryo transfer that a history of first trimester pregnancy loss following IVF has been reported to be a positive factor to predict future success with IVF treatment. They also showed that patients experiencing FTPL had significantly more top quality and cryopreserved embryos than patients with an ongoing pregnancy and thus should not be considered a poor prognosis group [6].

Levy et al also showed that after a biochemical pregnancy, the pregnancy outcome had better ongoing pregnancy rates (24.7%) in comparison with the 17% achieved in the total IVF-ET cycles [20].

Pearson et al found that among 2245 women who had IVF-ET, those who experienced a chemical pregnancy that failed to progress to a clinically recognized pregnancy or a spontaneous abortion on their first IVF cycle were more likely to discontinue IVF treatment than those whose first cycle ended prior to embryo transfer or who did not have a positive pregnancy test following transfer. However, among women who did continue to a second IVF cycle, those who had at least a chemical pregnancy on the first cycle were more likely to have a live birth on the second attempt than those women who had failed prior to conception in the first cycle (34% success rate compared to 21%, respectively) [21].

Bates et al determined the significance of biochemical pregnancy losses and clinical spontaneous abortion on outcomes of future IVF cycles in their unit in Boston through a retrospective cohort study. This involved women with a history of unsuccessful IVF attempts undergoing IVF. The main outcome measure was clinical pregnancy rate. Their results showed that patients with an early pregnancy loss had a greater ongoing clinical pregnancy rate in the immediate next cycle when compared with those women who had a negative pregnancy test (37.3% vs. 27.3%). Patients with a history of a biochemical pregnancy or a clinical spontaneous abortion had an ongoing clinical pregnancy rate in the next cycle of 38.4% and 42.3%, respectively, compared with 27.3% in women who had a history of a negative pregnancy test. The cumulative pregnancy rate after the first IVF attempt was 54.1% in patients with a previous biochemical pregnancy loss, 61.4% in those with a previous clinical spontaneous abortion and 46.5% in women with a previous negative pregnancy test. They concluded that women who experience an early pregnancy loss after IVF have a greater likelihood of success in subsequent IVF cycles when compared with patients who fail to conceive [22].

Additionally, Weckstein et al investigated the significance of a biochemical pregnancy in an IVF cycle in terms of its prognostic importance for a successful pregnancy in subsequent IVF cycles. They retrospectively evaluated biochemical pregnancies arising from IVF cycles, and pregnancy outcome in subsequent cycles. They compiled data from all IVF cycles between Jan 1998 and August 2000. They analysed patients having a biochemical pregnancy during that period for cycle outcome in other IVF cycles. Biochemical pregnancy was defined as hCG levels > 5 on 2 occasions 15days or greater after hCG injection, with no gestational sac ever visible with ultrasound. Their results showed 67 patients had biochemical pregnancy, 33 of these patients did not undergo any further IVF cycles, 34 patients underwent 1 or more subsequent cycles, 20 of these (59%) subsequently delivered or had an ongoing pregnancy beyond 12 weeks. In 17 patients, the delivery resulted from the IVF cycle immediately following the biochemical pregnancy, and in 3 patients the delivery resulted from second IVF cycle following the biochemical pregnancy, 2 patients (6%) had subsequent clinical pregnancy losses and 12 (35%) had negative cycles after the biochemical pregnancy (two of these had more than one subsequent biochemical pregnancy). They concluded from their preliminary data that a biochemical pregnancy is not indicative of a poor prognosis for future IVF cycles. Patients who have a biochemical pregnancy should be encouraged to go through another IVF cycle [23].

## Treatment of a Chemical Pregnancy

No specific treatment is required for a biochemical pregnancy. The most important follow-up test is to ensure that the hCG levels decline to non-detectable levels in order to differentiate it from an ectopic pregnancy. In order to minimize psychological morbidity and address patients concerns, a post-treatment follow-up appointment is a ‘sine qua non’. Armed with the evidence of a better prognosis for subsequent treatment cycle, the patient should be reassured that there is no adverse impact on future pregnancies and the prognosis for future fertility remains good.

## Conclusion

In conclusion, 1). The occurrence of a biochemical pregnancy after assisted conception leaves a lot of unanswered questions in the mind of the patient and is a cause of psychological morbidity. 2). The exact aetiology is unknown though several attributable factors have been proposed. 3). However, the occurrence of a biochemical pregnancy provides some light at the end of the tunnel as the patient can be advised about the optimistic outcome for subsequent treatment cycles. Patient support is paramount. 4). The onus lies on ART practitioners to explore further for answers as to predictive factors for a biochemical pregnancy.

## Abbreviations

IVF: In Vitro Fertilisation
ART: Assisted Reproductive Technology
β-HCG: Beta Human Chorionic Gonadotropin
ET: Embryo Transfer
ICSI: Intracytoplasmic Sperm Injection
